# A previously uncharacterized *O*-glycopeptidase from *Akkermansia muciniphila* requires the Tn-antigen for cleavage of the peptide bond

**DOI:** 10.1016/j.jbc.2022.102439

**Published:** 2022-08-30

**Authors:** Brendon J. Medley, Leif Leclaire, Nicole Thompson, Keira E. Mahoney, Benjamin Pluvinage, Matthew A.H. Parson, John E. Burke, Stacy Malaker, Warren Wakarchuk, Alisdair B. Boraston

**Affiliations:** 1 Department of Biochemistry and Microbiology, University of Victoria, Victoria, British Columbia, Canada; 2 Department of Biological Sciences, University of Alberta, Edmonton, Canada; 3 Department of Chemistry, Yale University, New Haven, Connecticut, USA; 4 Department of Biochemistry and Molecular Biology, University of British Columbia, Vancouver, British Columbia, Canada

**Keywords:** protease, *O*-glycoprotease, *O*-glycopeptidase, mucinase, metallopeptidase, *Akkermansia muciniphila*, mucin, microbiome, ACN, acetonitrile, AGC, automatic gain control, BSM, bovine submaxillary mucin, CBM, carbohydrate-binding module, HCD, higher energy collisional dissociation, HDX-MS, hydrogen-deuterium exchange mass spectrometry, IgA, immunoglobulin A, UPLC, ultraperformance liquid chromatography

## Abstract

*Akkermansia muciniphila* is key member of the human gut microbiota that impacts many features of host health. A major characteristic of this bacterium is its interaction with host mucin, which is abundant in the gut environment, and its ability to metabolize mucin as a nutrient source. The machinery deployed by *A. muciniphila* to enable this interaction appears to be extensive and sophisticated, yet it is incompletely defined. The uncharacterized protein AMUC_1438 is encoded by a gene that was previously shown to be upregulated when the bacterium is grown on mucin. This uncharacterized protein has features suggestive of carbohydrate-recognition and peptidase activity, which led us to hypothesize that it has a role in mucin depolymerization. Here, we provide structural and functional support for the assignment of AMUC_1438 as a unique *O*-glycopeptidase with mucin-degrading capacity. *O*-glycopeptidase enzymes recognize glycans but hydrolyze the peptide backbone and are common in host-adapted microbes that colonize or invade mucus layers. Structural, kinetic, and mutagenic analyses point to a metzincin metalloprotease catalytic motif but with an active site that specifically recognizes a GalNAc residue α-linked to serine or threonine (*i.e.*, the Tn-antigen). The enzyme catalyzes hydrolysis of the bond immediately N-terminal to the glycosylated residue. Additional modeling analyses suggest the presence of a carbohydrate-binding module that may assist in substrate recognition. We anticipate that these results will be fundamental to a wider understanding of the *O*-glycopeptidase class of enzymes and how they may contribute to host adaptation.

The mammalian gastrointestinal tract is protected by a mucosal barrier that has inner and outer layers. The dense inner layer is tightly adhered to epithelial cells and effectively bacteria free, whereas the outer layer is a loose matrix that is colonized by bacteria (see Ref. (1) for a review). Both layers largely comprise mucins, densely *O-*glycosylated proteins that have 60 to 70% complex carbohydrate chains by weight, with the inner layer having membrane-associated mucins and the outer layer having unattached gel-forming mucins. The diverse carbohydrate chains of mucin promote colonization of bacterial species that possess the proper metabolic capabilities to forage for glycans as a nutrient source.

*Akkermansia muciniphila* is a Gram-negative bacterium of the phylum Verrucomicrobia (2). It is a common gastrointestinal commensal in animals and found comprising 3 to 5% of a healthy human gut microbiota (3). Since the initial description of this bacterium (2), its abundance in the human microbiome has been correlated with an enormous array of healthy or disease states, highlighting the importance of its role in gut homeostasis and overall host health (see Ref. (4) for an overview).

*A. muciniphila* is known for its capacity to deconstruct and utilize mucin as a nutrient source. Genomic analysis of this bacterium combined with transcriptomic studies when grown on mucin indicate that approximately 3% of the genes in the *A. muciniphila* genome contribute to mucin degradation (5, 6). A large number of these genes are carbohydrate-active enzymes devoted to carbohydrate processing, which reflects the abundance of glycans in mucins. However, notable amongst the mucin-processing enzymes are three proteins classified in the MEROPS satabase (7) as family M60 peptidases (AMUC_0627, AMUC_0908, and AMUC_2001), one that is classified as M98 (AMUC_1514), and the unclassified metzincin-like peptidase OgpA (AMUC_1119). The *amuc_0627*, *amuc_0908*, and *amuc_2001* genes are upregulated when *A. muciniphila* is grown on mucin, supporting their role in mucin metabolism, whereas AMUC_0627, AMUC_0908, AMUC_1514, and OgpA have demonstrated *in vitro* mucinase activity (8, 9). These proteins belong to the clan MA of metallopeptidases, and all share the properties of requiring the recognition of an *O*-linked glycan on the substrate and cleavage of the peptide bond near the site of glycosylation, typically immediately N-terminal to the glycosylated residue (10). There are now several examples of these so-called *O-*glycopeptidases, *O*-glycoproteases, or mucinases, all of which originate from host-adapted microbes (11, 12).

Amongst the *A. muciniphila* genes upregulated when the bacterium is grown on mucin is one that encodes AMUC_1438. The encoded protein is annotated as “glycosyl hydrolase family 98 putative carbohydrate-binding module” by virtue of an easily identifiable C-terminal module of ∼140 amino acids that is annotated as belonging to Pfam family PF08305 (NPCBM/NEW2 domain). The N-terminal domain is annotated as a “metallopeptidase,” likely because of the presence of an HEXXH motif (13). However, this domain is not clearly identified with any particular domain family as only ∼30 amino acids surrounding the metallopeptidase motif are classified into PF12044. On the basis of these observations for AMUC_1438—upregulation on mucin, possible carbohydrate-binding function, and possible peptidase activity—we hypothesized that this protein is an *O*-glycopeptidase that falls into an as yet uncharacterized group of metallopeptidases.

We examined the function of AMUC_1438 through domain dissection, structural studies, and activity assays on a variety of glycoproteins and *O*-glycosylated peptides. The results reveal that the enzyme is an *O*-glycopeptidase that cleaves immediately at the N-terminal side of serine or threonine residues bearing a single α-linked *O*-GalNAc residue, that is, the Tn-antigen. The catalytic domain of AMUC_1438 belongs to the “metzincin” class of peptidases and is structurally related to OgpA from *A. muciniphila*, though they are only distantly related at the amino acid sequence level (<25% identity). The specificity of OgpA is similar to that of the known M60-like peptidases from *A. muciniphila* (AMUC_0627, AMUC_0908, and AMUC_1514), which accept a variety of linear *O*-glycans (8). The strict specificity AMUC_1438 is therefore unique amongst currently characterized *O*-glycopeptidases, including known mucin-degrading enzymes of *A. muciniphila*, providing new insight into the interaction of *A. muciniphila* with its environment in the host.

## Results

### Dissection of AMUC_1438 and activity on mucin

BLAST (14) queries with AMUC_1438 did not reveal any sequence identity with functionally characterized proteins. Analysis of the amino acid sequence with InterProScan indicated the presence of a secretion signal peptide (amino acids 1–26) and an unidentified region (amino acids 27 to ∼498) containing an HEXXH metallopeptidase motif (13, 15). The ∼30 amino acids surrounding this motif are indicated to belong to an “uncharacterized protein family, zinc metallopeptidase-like” (PF12044 and IPR021917). Though the unidentified region contained a metallopeptidase motif, BLAST searches against the MEROPS database returned no hits with classified peptidase families (7). Searches of the C-terminal NPCBM domain of AMUC_1438 (amino acids ∼499–639) against the dbCAN Meta Server using the HMMER function indicated potential identity with carbohydrate-binding module (CBM) family 51 (16), which is consistent with the content of the characterized modules in PFam family PF08385 to which the module belongs. However, this module is categorized in the Carbohydrate-Active Enzyme Database (CAZyDB) as belonging to an unclassified CBM family (17).

**Figure 1:**
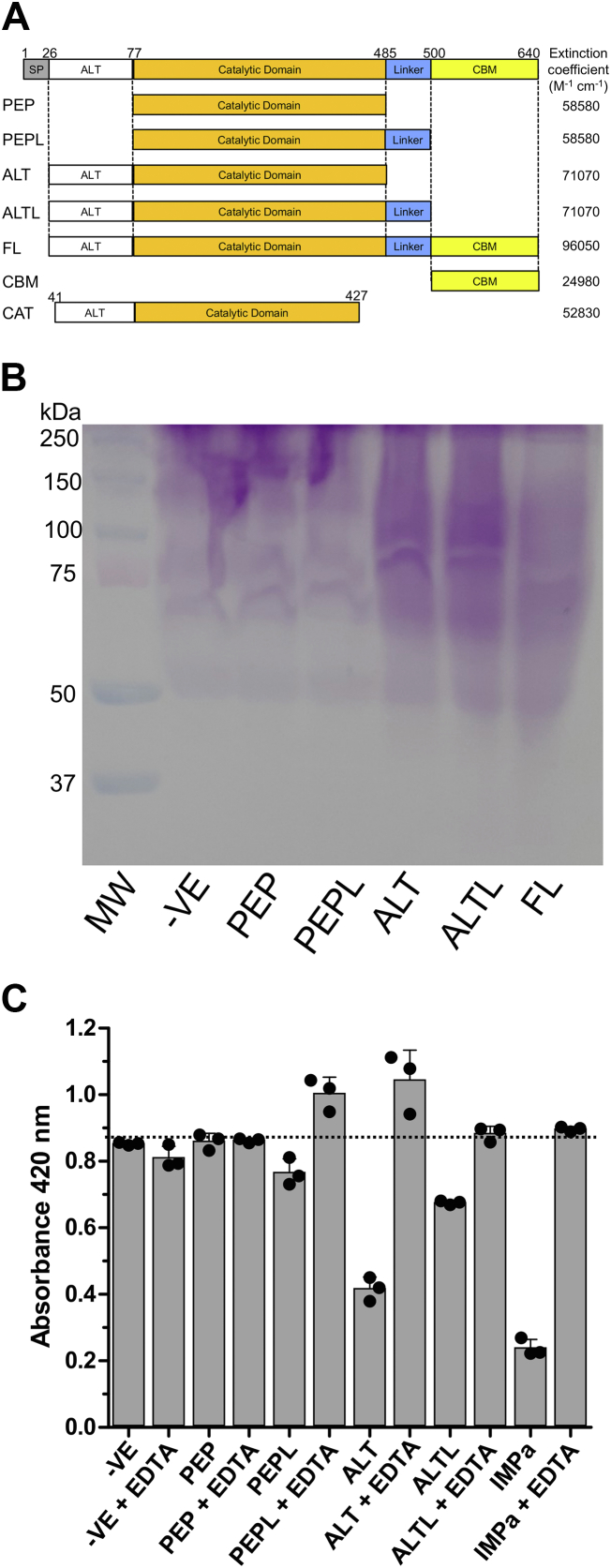
**Schematics and activities of the AMUC_1438 truncated constructs used in this study.***A*, amino acid numbering for the construct boundaries is shown above for all constructs. Names identifying the proteins are given to the *left* of the specific constructs. Calculated extinction coefficients are given to the *right* of the specific constructs. *B*, activity of the AMUC_1438 truncated constructs. *A*, an SDS-PAGE gel developed with periodic acid-Schiff stain for carbohydrates. The negative (-VE) control is untreated BSM. Remaining lanes were treated with the AMUC_1438 construct indicated in the *lane label*. *C*, microtiter plate–based mucinase assay (10). The -VE control is untreated BSM or treated with only EDTA. Remaining samples were treated with the AMUC_1438 construct indicated in the label, with or without EDTA as indicated. Activity on BSM is indicated by a decreased absorbance resulting from removal of biotinylated BSM from the plate surface by enzyme degradation. IMPa is provided as a positive control. Error bars indicate the standard deviation of three independent replicates; individual data points are also shown. BSM, bovine submaxillary mucin.

To enable a functional analysis of AMUC_1438, we generated several truncated versions derived from our bioinformatics analysis for recombinant expression in *Escherichia coli*. These were called PEP, PEPL, ALT, ALTL, FL, and CBM with their domain boundaries outlined in Figure 1*A*. We were unable to produce soluble CBM, and PEP proved to be very unstable, though we still included it in our initial assays. The remaining constructs were soluble, stable, and could be purified to >95% homogeneity as judged by SDS-PAGE (Fig. S1).

Given the presence of the metallopeptidase motif in the PEP, PEPL, ALT, ALTL, and FL constructs, we initially assessed these for generic peptidase activity using a commercial casein-based assay. This failed to reveal any activity. Based on the upregulation of *amuc_1438* expression when *A. muciniphila* is grown on mucin, and the presence of a putative CBM, we postulated that the protein may have activity on mucins. ALT, ALTL, and FL, but not PEP or PEPL, appeared to cause a change in the mobility of bovine submaxillary mucin (BSM) in SDS-PAGE gels after treatment with the proteins (Fig. 1*B*). This suggested activity on BSM, which we then supported by a microtiter plate–based mucinase assay where mucin degradation is visualized as the loss of immobilized biotin-labeled BSM from the plate (Fig. 1*C*). None of the proteins appeared to display activity on asialofetuin or fetuin (not shown).

**Figure 2:**
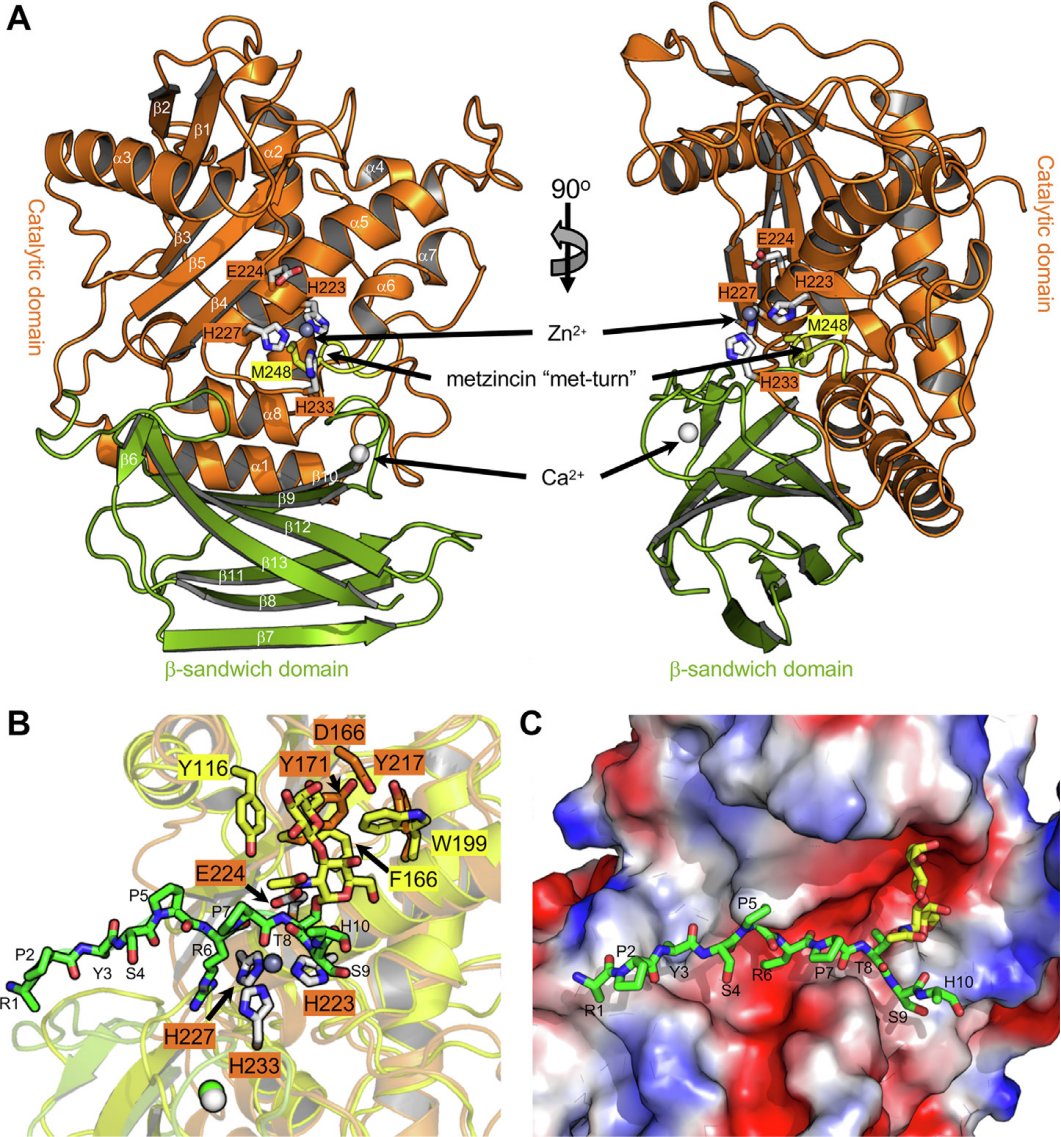
**Structural analysis of AMUC_1438 by X-ray crystallography.***A*, a *cartoon* representation of the 2.35 Å resolution structure of the ALT construct. The structure is colored by domain with the zinc-binding catalytic center shown as *gray sticks* and the metzincin turn colored in *yellow*. *B*, an overlap of the ALT construct (*orange* and *gray*) with the structure of OgpA (*yellow*) in complex with glycodrosocin (*green* and *yellow sticks* for the peptide and glycan, respectively) (Protein Data Bank ID: 6Z2P). *C*, solvent accessible surface representation of ALT with the glycodrosocin peptide retained from the overlap with OgpA. The surface is colored according to electrostatic potential from *blue* (positive) to *red* (negative).

### Structural analysis of AMUC_1438

The structure of ALT was determined by single isomorphous replacement with anomalous scattering using a native dataset and a derivative obtained by soaking a crystal in sodium iodide (see Table 1 and Experimental procedures section). The structure determined to 2.35 Å contained four protein molecules in the asymmetric unit, all of which were missing ∼60 amino acids at C terminus that could not be modeled.

**Table 1:** X-ray data collection and structure statistics

	ALT iodide	ALT native	ALTL
*Data collection*			
Beamline	Home beam	Home beam	Home beam
Wavelength (Å)	1.54178	1.54178	1.54178
Space group	P2_1_2_1_2_1_	P2_1_2_1_2_1_	P2_1_2_1_2_1_
Cell dimensions			
*a*, *b*, *c* (Å)	88.7, 145.8, 147.5	88.6, 146.1, 147.3	71.2, 91.5, 161.7
α, β, γ (º)	90.0, 90.0, 90.0	90.0, 90.0, 90.0	90.0, 90.0, 90.0
Resolution (Å)	25.00–2.40 (2.44–2.40)	25.00–2.35 (2.39–2.35)	25.00–2.50 (2.54–2.50)
*R*_meas_	0.153 (1.131)	0.159 (0.393)	0.092 (0.346)
*R*_pim_	0.030 (0.339)	0.072 (0.228)	0.043 (0.195)
CC1/2	0.998^a^ (0.715)	0.986^a^ (0.902)	0.992^a^ (0.860)
<I/σI>	24.7 (2.0)	8.2 (1.9)	15.2 (2.8)
Completeness (%)	99.8 (97.6)	98.2 (96.9)	98.7 (96.5)
Redundancy	24.8 (10.0)	3.8 (2.5)	4.1 (2.9)
No. of reflections	1,880,433	289,777	147,743
No. unique	75,769 (3634)	78,394 (3818)	36,634 (1773)
*Refinement*			
Resolution (Å)		25.00–2.35	25.00–2.50
*R*_work_/*R*_free_		0.23/0.27	0.23/0.28
No. of atoms			
Protein		2800 (A), 2820 (B), 2809 (C), 2830 (D)	3265 (A), 3292 (B)
Ligand		4 Zn, 4 Ca	2 Zn
Water		320	60
*B*-factors			
Protein		33.1 (A)/33.7 (B)/36.8 (C)/34.2 (D)	34.4 (A), 40.2 (B)
Ligand		37 (Zn), 34.4 (Ca)	41.2
Water		30.7	27.8
RMSD			
Bond lengths (Å)		0.002	0.002
Bond angles (°)		0.552	0.449
Ramachandran (%)			
Preferred		96.8	97.4
Allowed		2.9	2.6
Disallowed		0.3	0.0

Values for highest resolution shells are shown in parenthesis.

a Value refers to low-resolution shell.

The core fold of the protein comprises a five-stranded β-sheet with a single α-helix packed on one face and region of multiple α-helices on the other face of the β-sheet (Fig. 2*A*). Pressed against this α-helical region is a β-sandwich domain comprising opposing four-stranded and three-stranded antiparallel β-sheets. Bound to the β-sandwich domain was a metal ion that was modeled as a calcium atom. A central feature of this catalytically active *O*-glycopeptidase fold is an ∼30 Å long helix that contains the HEXXH portion of the zinc-binding motif where the glutamate is the catalytic residue (Fig. 2*A*). The third histidine residue outside the canonical metallopeptidase motif, H233, completes the zinc-binding motif and is found on a loop structurally adjacent to the α-helix supporting the rest of the catalytic machinery. Underlying this is the “met-turn,” which places a methionine side chain directly beneath the zinc-binding site. Together, these features identify the catalytic center as having a metzincin motif (Fig. 2*A*) (18). Overall, this fold is the same as that described for OgpA, the most structurally similar protein to ALT (RMSD of 2.1 Å, amino acid sequence identity of 19% over 292 aligned residues) and whose structure was described in detail previously (9) (Fig. S2).

We overlapped ALT with the structure of OgpA in complex with the *O*-glycopeptide glycodrosocin (Fig. 2*B*). The catalytic machinery, identified by the Zn^2+^-binding site and catalytic residue E224, overlapped almost perfectly. Apart from this conservation in the S1′ subsite, none of the residues involved in recognition of the peptide portion of glycodrosocin by OgpA were conserved in the other S subsites. However, two tyrosine residues in ALT, Y171 and Y217, were structurally conserved with a phenylalanine and a tryptophan in OgpA, both of which are structurally implicated in glycan recognition in the G′ subsites of OgpA (9). This comparison suggested that the tyrosine residues in AMUC_1438 may comprise an *O*-glycan-binding site in this enzyme and, overall, pointed to the enzyme being an *O*-glycopeptidase similar to OgpA. A surface representation of ALT with the glycodrosocin peptide from the OgpA overlap reveals the groove comprising the substrate-binding site (Fig. 2*C*). However, consistent with the lack of conservation in the P subsites, numerous clashes between the surface and the glycopeptide, particularly at residues 3, 5, and 6 of the peptide, indicate that the AMUC_1438 must accommodate substrate in a manner that is different from that of OgpA.

### Catalytic activity is dependent upon the presence of the Tn-antigen

**Figure 3:**
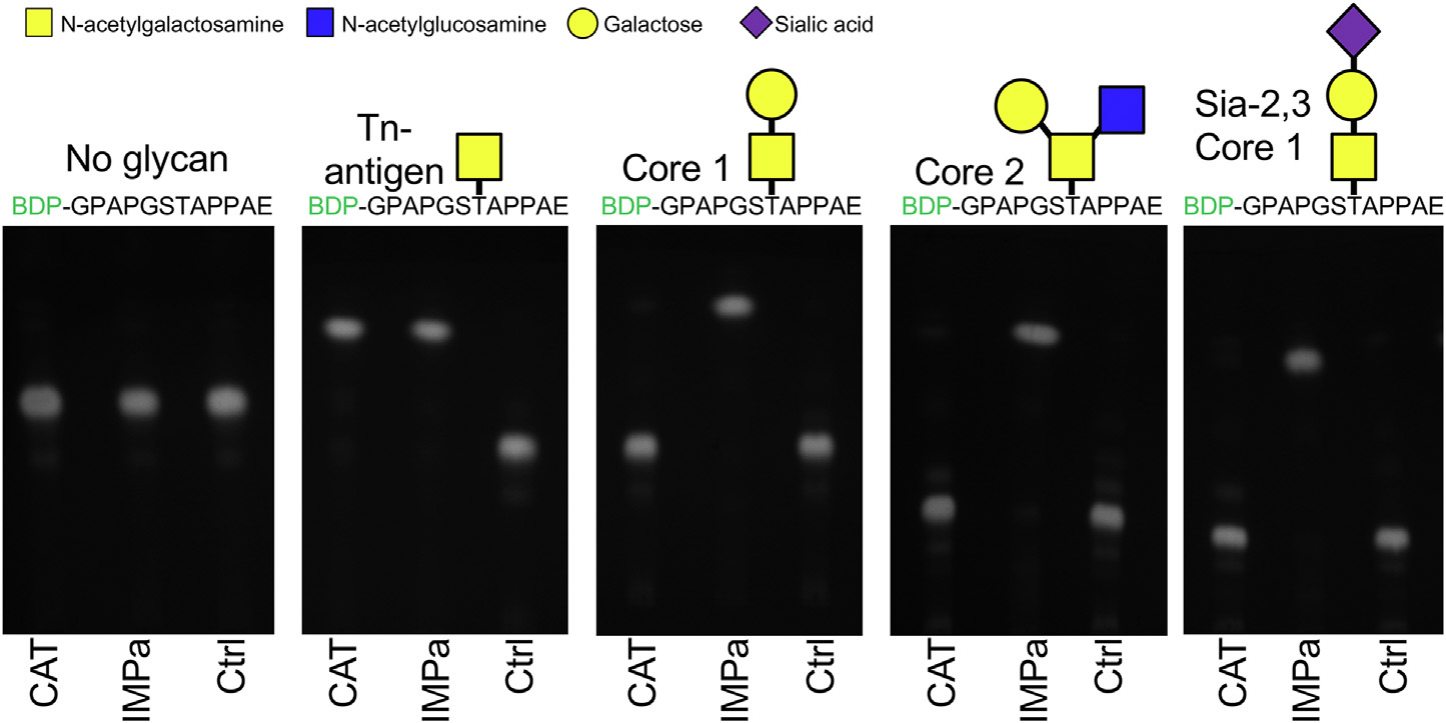
**TLC analysis of CAT activity on defined *O*-glycopeptides.** The identity of the *O*-glycopeptide is given above each *panel*. BDP indicates the BODIPY fluorophore tag. Ctrl is the untreated peptide; IMPa is the positive control. TLC plates were imaged under UV at a wavelength of 365 nm.

On the basis of the crystal structure of ALT, we generated an additional construct, CAT (Fig. 1*A*), with domain boundaries minimized for production of an active catalytic region. We used this construct to test our hypothesis that a truncation of AMUC_1438 possesses specific *O*-glycopeptidase activity. We did so by assessing the activity of CAT on a representative set of defined chemoenzymatically generated *O*-glycopeptides based on a MUC1 peptide (Fig. 3). CAT only displayed activity on the peptide bearing a single *O*-GalNAc (Tn-antigen) and no activity on the peptide completely lacking an *O*-glycan, thus demonstrating that the enzyme is an *O*-glycopeptidase. CAT had no activity on peptides with extended glycans indicating a strict requirement for the Tn-antigen. The positive control, IMPa, is a family M88 *O*-glycopeptidase of the M60-like superfamily. It has quite broad specificity for both peptide sequence and glycan structure that is known to cleave immediately N-terminal to site of *O*-glycosylation (10, 19). The product of CAT activity on the Tn-antigen peptide displayed similar mobility to that of the IMPa, thus also suggesting that CAT cleaved N-terminal to the site of *O*-glycosylation.

**Figure 4:**
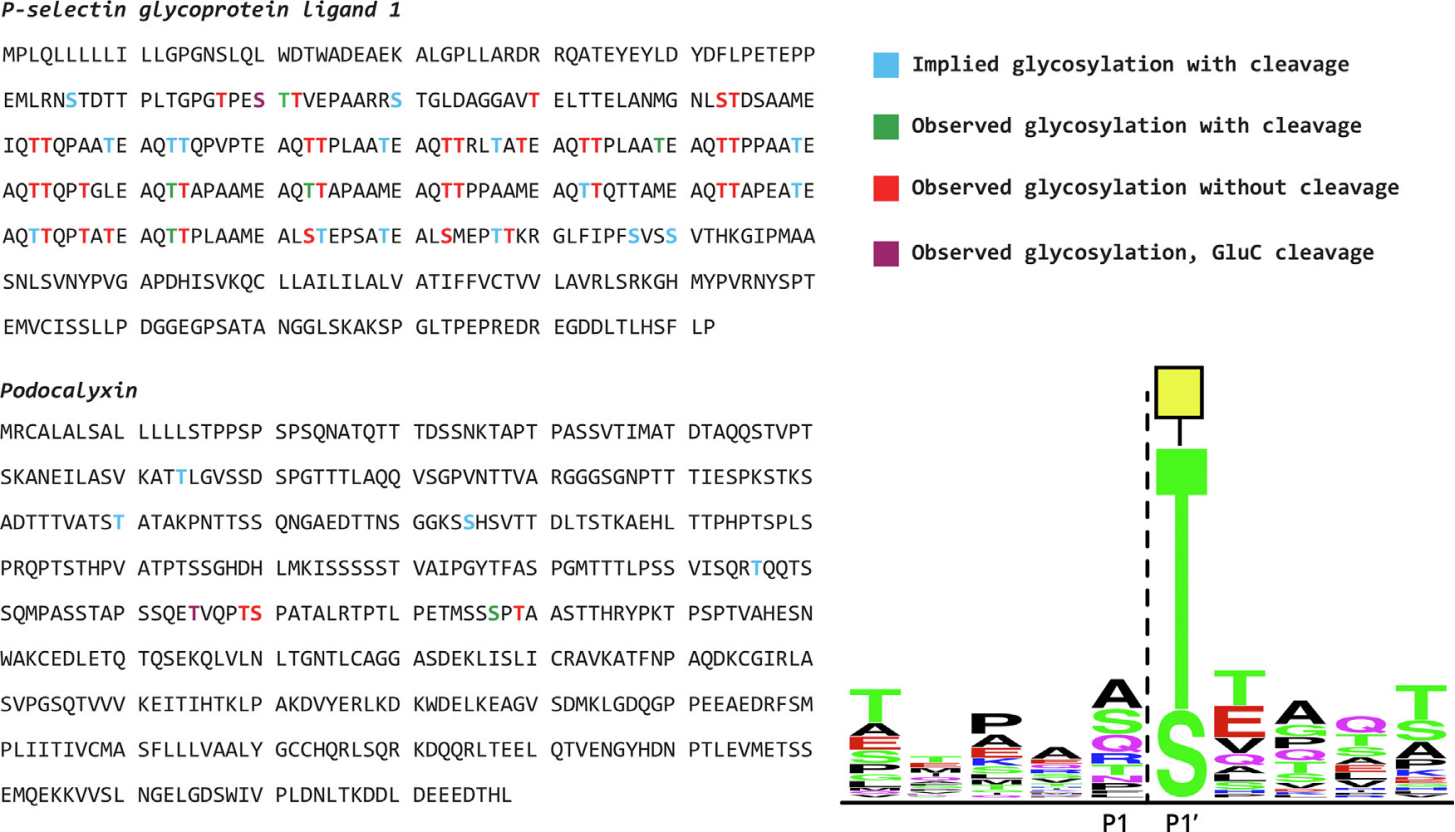
**Mapping of AMUC_1438, CAT construct, cleavage in recombinant mucin–domain glycoproteins.** Peptides present in the CAT-treated samples were used as input for weblogo.berkeley.edu (±5 residues from the site of cleavage). The consensus motif of CAT is representative of 42 unique cleavage sites.

**Figure 5:**
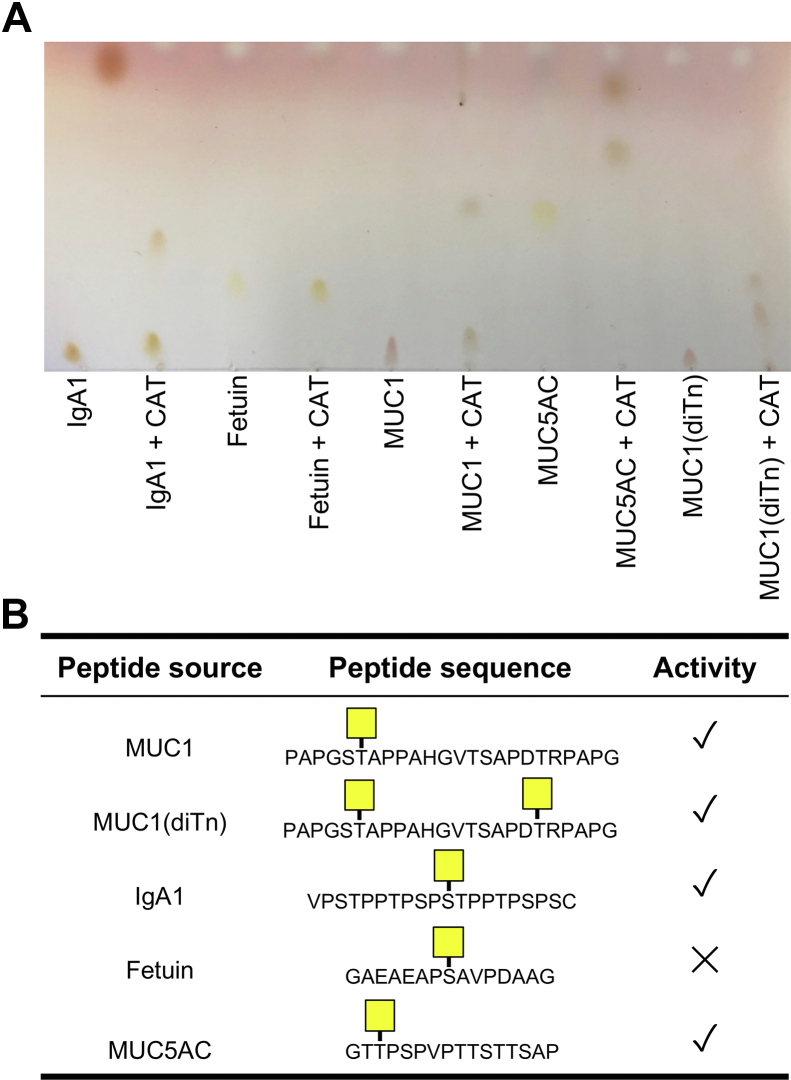
**Cleavage of different *O*-glycopeptides bearing the Tn-antigen.***A*, TLC separation of *O*-glycopeptides treated with CAT. *B*, summary of the results and structures of the peptides used in the TLC.

To further support this, using a previously established mass spectrometric methodology (8, 20), we mapped the cleavage sites of CAT in a selection of representative mucin-like glycoproteins. All of the detected cleavage sites were immediately N-terminal to *O*-glycosylation sites bearing the Tn-antigen, providing confirmation of the results with the peptides (Fig. 4). Cleavage sites were relatively infrequent in comparison to the known abundance of *O*-glycosylation sites on the substrates, likely reflecting the paucity of the Tn-antigen in the glycoprotein substrates. Other than the requirement for the Tn-antigen, the cleavage site mapping results did not clearly reveal any potential preference for the amino acid sequence surrounding the site of *O*-glycosylation. However, we also screened activity on five specific Tn-antigen bearing peptides and this showed that CAT was not active on a peptide sequence derived from fetuin, indicating an as yet undefined dependence of activity on aspects of the amino acid sequence of the substrate (Fig. 5, *A*, *B*).

### Kinetic analysis of *O*-glycopeptidase activity

**Figure 6:**
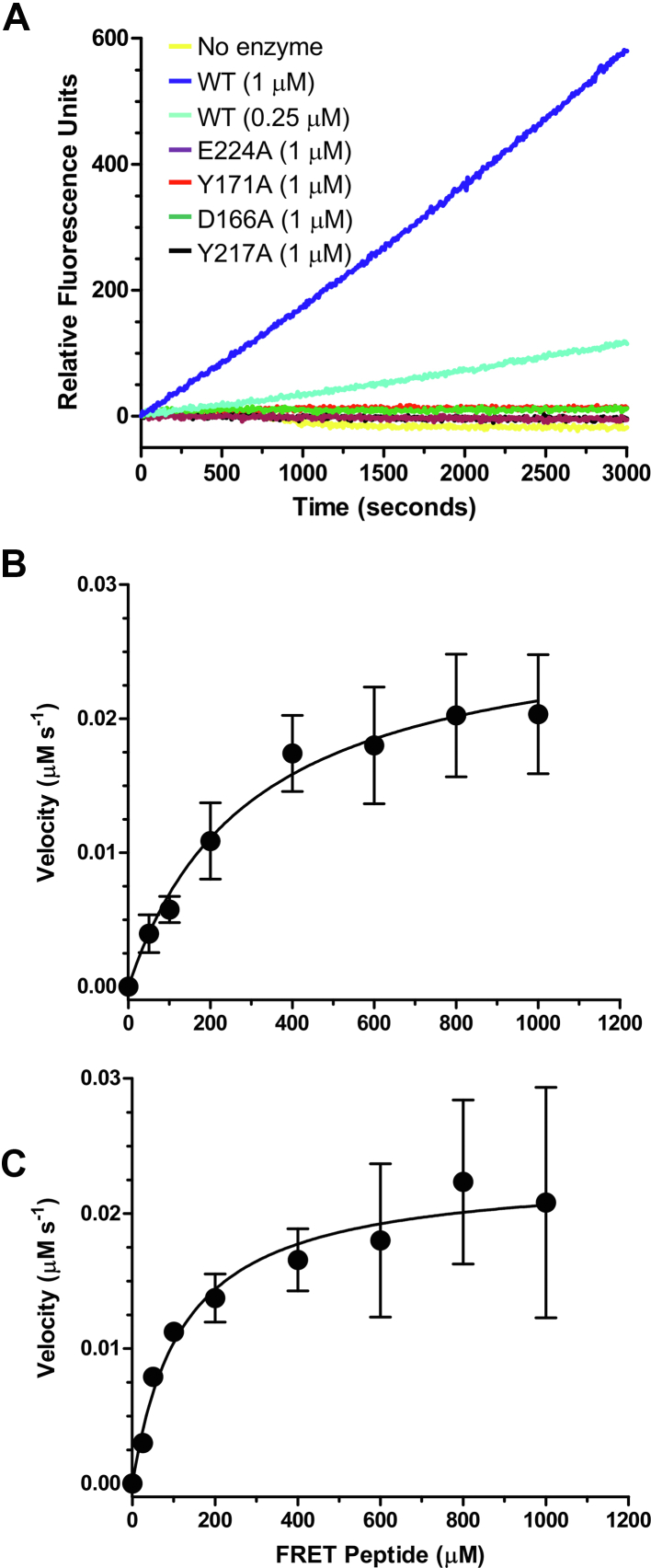
**Kinetic analysis of AMUC_1438 truncations and mutants on a FRET-based *O*-glycopeptide substrate.***A*, rates of FRET substrate cleaved for CAT and mutants of CAT. *B* and *C*, Michaelis–Menten plots for CAT and FL constructs. Error bars indicate the standard deviations of six independent replicates. The *solid line* shows the best fit to the Michaelis–Menten equation. Individual data points for the plots are shown in Figure 3.

To quantify AMUC_1438 *O*-glycopeptidase activity, we created a FRET assay (see the Experimental procedures section for details). The substrate was based on an immunoglobulin A (IgA)-hinge peptide with the sequence TPSP**S**TPPTK where the bold and underlined serine bears the α-linked *O*-GalNAc residue. Cleavage of the substrate gave strong dequenching of fluorescence in a manner dependent upon time and CAT concentration (Fig. 6*A*), and substrate concentration, allowing quantification of hydrolysis kinetics by purified CAT and FL proteins (Figs. 6, *B*, *C*, and S3). CAT displayed a *K*_*M*_ of 300 (±70) μM and a *k*_cat_ of 1.7 (±0.2) min^−1^. The corresponding values for FL were 122 (±30) μM and 1.4 (±0.1) min^−1^.

The superimposition of the ALT structure with OgpA suggested a possible role of Y171 and Y217 in *O*-GalNAc recognition. In addition, a neighboring aspartic acid, D166, seemed a potential candidate for a hydrogen-bonding role. We were unable to generate cocrystal structures of any of our AMUC_1438 constructs; so, to test this hypothesis, we generated alanine substitutions of the three residues and examined their activity on the IgA-hinge FRET peptide. We used an E224A mutant of the catalytic residue as an inactive negative control. Though the mutants retained their stability, as assessed by differential scanning fluorimetry (Fig. S4), they displayed no activity on the FRET peptide (Fig. 6*A*). This supports the role of all three residues in substrate recognition, most likely *via* the proposed interactions with the *O*-linked GalNAc residue.

### Modeling of full-length AMUC_1438

**Figure 7:**
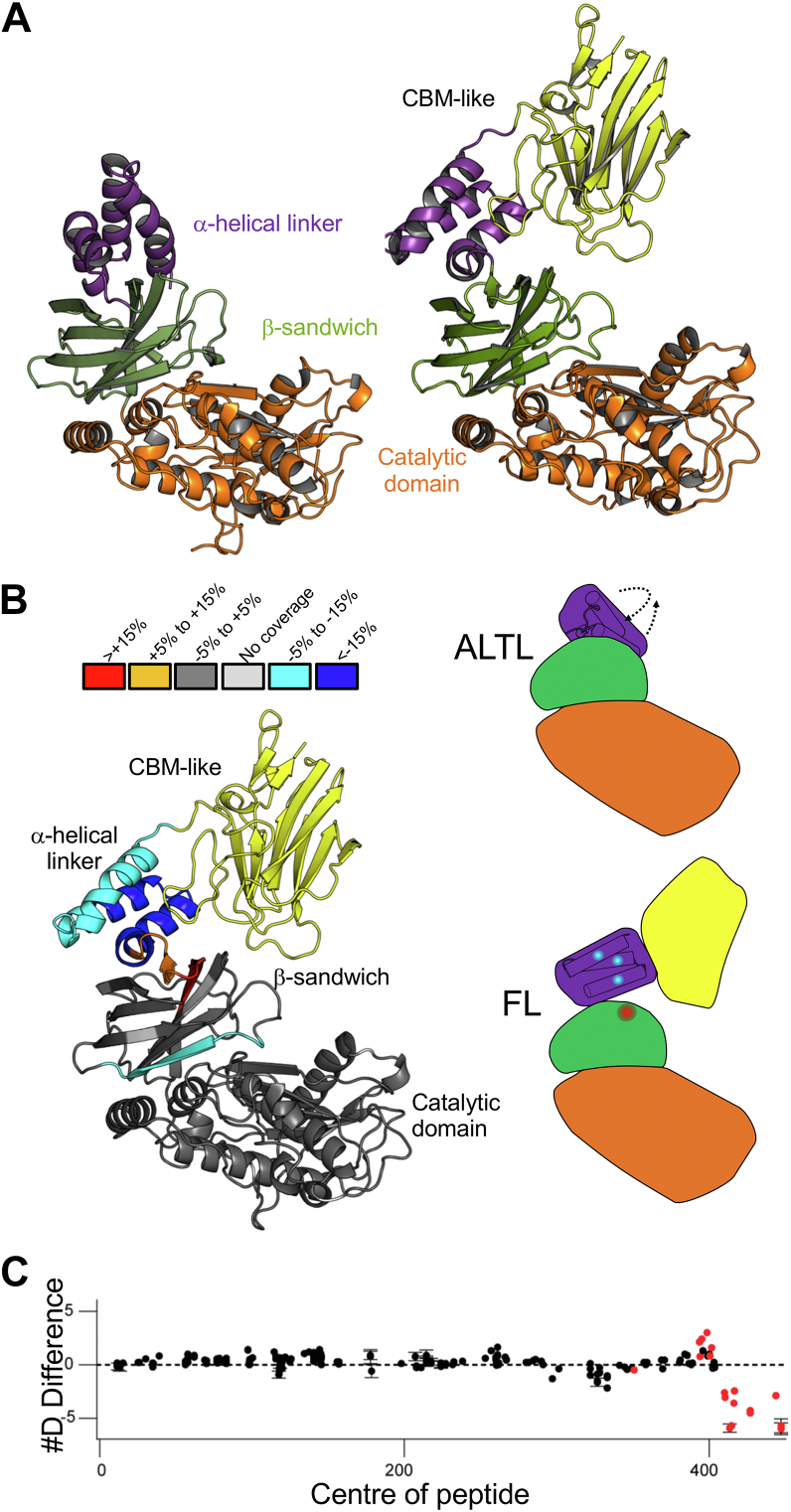
***In silico* and HDX-MS analysis of the AMUC_1438 structure.***A*, *cartoon* representations of the 2.5 Å resolution X-ray crystal structure of the ALTL construct (*left*) and an AlphaFold2-generated model of the FL structure (*right*). *B*, conformational analysis of ALTL and FL by HDX-MS results. Maximum significant HDX differences in FL relative to ALTL were observed across all time points and mapped on the FL AlphaFold2 model, with the exception of the CBM, which is lacking in ALTL protein (*right*). Color represents the presence of significant differences in exchange with the relevant peptides colored by percent change between the two proteins according to the legend immediately above the image. Conformational differences between FL and ALTL with changes in HDX are schematically shown on the *right*. *Arrows* indicate the directions the α-helical domain moves between the ALTL conformation and the FL conformation. Coloring of domains in the schematics is the same as that used in *A*. *C*, deuterium incorporation difference between selected peptides (based on the truncated ALTL sequence) that showed a significant increase or decrease in exchange between the FL and ALTL constructs (>5%, 0.4 Da, and an unpaired *t* test, *p* < 0.01). For all panels, error bars show SD (n = 3). CBM, carbohydrate-binding module; HDX-MS, hydrogen-deuterium exchange mass spectrometry.

Toward establishing structure–function relationships for the full-length multimodular AMUC_1438 protein, we attempted to crystallize larger fragments of the protein. We were unable to generate crystals of FL, but we were able to determine the structure of ALTL to 2.5 Å resolution. In this case, the last 18 amino acids in the two molecules in asymmetric unit were missing. The last ∼60 C-terminal amino acids that could be modeled, and which correspond to the missing residues in the ALT structure, comprised a three α-helix bundle (Fig. 7*A*) resembling the Found In Various ARchitectures (FIVAR) domains observed as linkers in the ZmpB *O*-glycopeptidase from *Clostridium perfringens* (21).

We also modeled the FL construct using AlphaFold2 (Fig. 7*A*) (22). A comparison of the ALT structure to the catalytic domain of the FL model yielded an RMSD of 1.3 Å. A similar comparison focusing only on the all α-helical linker from the ALTL structure gave an RMSD of 0.7 Å. Overall, this reveals the remarkable accuracy of the AlphaFold2 model with respect to the domains for which we have experimental structures, thereby giving confidence in the model of the uncharacterized CBM-like domain. However, the relative orientations of the α-helical linker domain and the catalytic domain in the ALTL structure differ from the FL model, with the α-helical linker being packed more closely against the β-sandwich domain in ALTL than in the FL model (Fig. 7*A*). Indeed, the conformation observed in ALTL seems unlikely in the full-length enzyme as the position of the α-helical linker domain would likely result in clashes between the CBM-like domain, if it were present, and the rest of the protein.

Toward providing support for the conformation observed in the FL model compared with the ALTL structure, we employed hydrogen-deuterium exchange mass spectrometry (HDX-MS) on both proteins. A comparison of detected peptides showing significant differences in exchange in FL relative to ALTL, excluding the CBM that is absent in ALTL, showed the differences to be isolated largely in and around the α-helical linker domain, approximately residues 390 to 456 (Figs. 7, *B*, *C* and S5). In particular, HDX was decreased in the α-helical linker of FL relative to ALTL, suggesting that it is in some way more structured in FL, possibly through more compact folding and/or additional intradomain contacts with the CBM. Most relevant to the FL model, however, is the increased exchange in the last two β-strands of the β-sandwich domain and the initial 1 to 2 turns of the first α-helix in the α-helical linker domain. In the ALTL crystal structure, these secondary structures are shielded from solvent by packing of the α-helical linker against the β-sandwich domain. In the FL model, however, the α-helical linker is peeled away from the β-sandwich domain, thus exposing the protein regions in FL that were observed to exchange more readily. Therefore, the HDX-MS results are consistent with conformation of the FL model.

**Figure 8:**
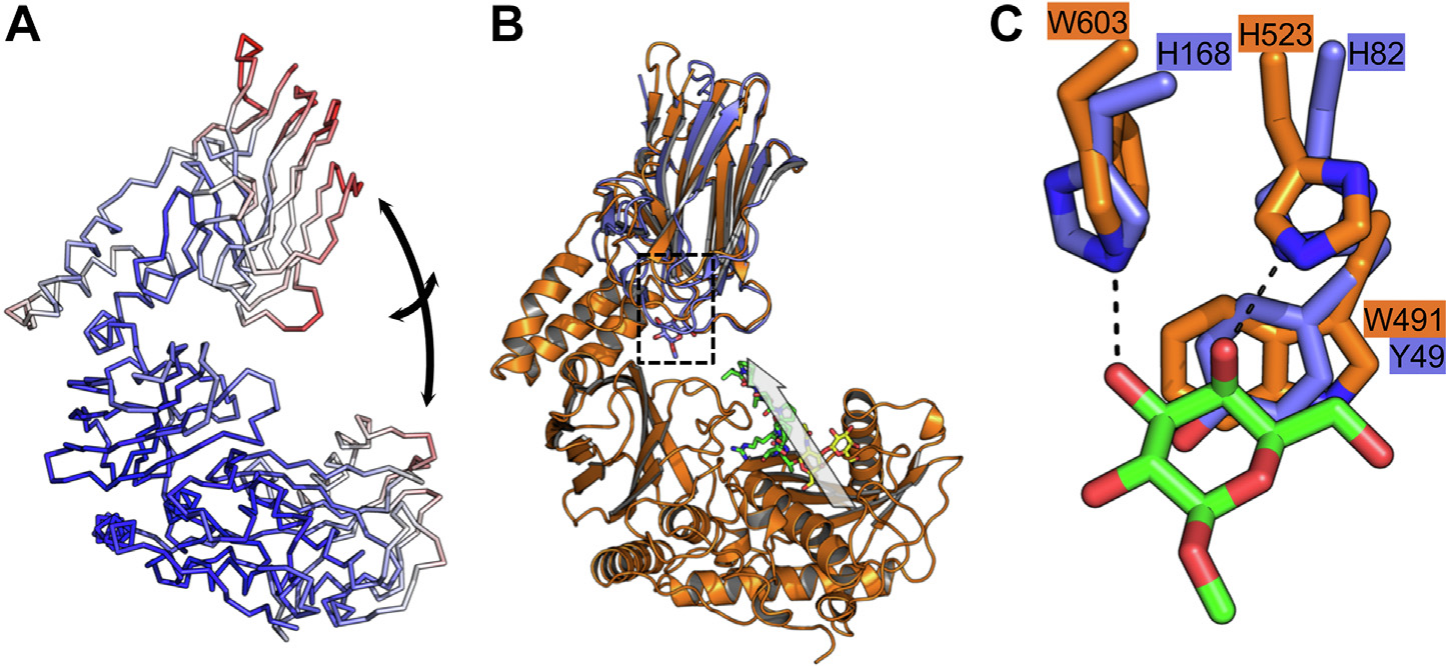
**Molecular motions and potential mode of substrate recognition by full-length AMUC_1438.***A*, normal mode analysis of the FL AlphaFold2 model performed with iMODS (45). Mobility is color ramped from low mobility to high mobility as *blue-white-red*. The *arrows* represent opening/closing and twisting motions of the CBM relative to the CAT domain. *B*, structure of the FL AlphaFold2 model (*orange*) overlapped with GH95CBM51 in complex with β-d-*O*-methyl galactose (*blue*, Protein Data Bank [PDB] ID: 2VMG). The structure of OgpA in complex with glycodrosocin (PDB ID: 6Z2P) was overlapped with the CAT region and the glycodrosocin (*green* and *yellow sticks* for the peptide and glycan, respectively) retained in the image to approximate potential substrate binding in AMUC_1438. The *gray arrow* represents the trajectory of the peptide in the direction of C to N terminus. *C*, the galactose-binding site in GH95CBM51 (*blue*) with bound ligand (*green*) and the conserved residues in the putative CBM of the FL model (*orange*). CBM, carbohydrate-binding module.

A normal mode analysis of the FL model did not indicate large molecular motions but did suggest some flexibility of this proposed conformation *via* opening–closing and twisting motions of the putative CBM relative to the catalytic module (Fig. 8*A*). Modeled movements were on the order of single-digit Angstrom distances. Highest deformability was computed in the region of the α-helical linker, suggesting that it acts as a hinge for the potential intradomain molecular motions (Fig. S6).

The CBM in the FL model displays the β-sandwich fold predicted by its similarity to CBM family 51 members. To date, three different modes of carbohydrate recognition have been observed in family 51 CBMs (21, 23). The model of the AMUC_1438 CBM displays significant structural similarity, including the binding site, to the galactose binding CBM51 from the *C. perfringens* family 95 glycoside hydrolase, GH95CBM51 (Fig. 8*B*) (23). The main tyrosine platform in GH95CBM51 is functionally conserved as a tryptophan in the putative AMUC_1438 CBM (Fig. 8*C*). Only one of the hydrogen-bonding histidines is conserved; however, the indole nitrogen of a tryptophan in the putative AMUC_1438 CBM is suitably placed to potentially hydrogen bond, thus functionally replacing the second histidine. Overall, the major galactose-recognition features of GH95CBM51 appear to be functionally conserved in the putative AMUC_1438 CBM, suggesting this putative CBM may have a role in glycan recognition.

## Discussion

The catalytically active region of AMUC_1438, defined by the CAT construct (residues 41–427), has amino acid sequence features that identify it as a metallopeptidase. This was corroborated by the structural analysis of the protein, which revealed a metzincin catalytic center, and mucinase activity that was inhibited by the metal chelator EDTA. However, other than the short region of sequence around the metal-binding site, this catalytic domain does not strongly associate with any defined families of characterized proteins.

A BLAST search against the nonredundant National Center for Biotechnology Information database using the CAT region of AMUC_1438 as a query, with all *A. muciniphila* sequences filtered out, and using the cutoff criteria of a minimum 25% amino acid sequence identity and 60% sequence coverage, returns several hundred similar sequences (∼500 at present) from over 200 species of bacteria (Table S1). These sequences are roughly equally distributed over the phyla Verrucomicrobiota, Planctomycetota, and Bacteroidota. A survey of the source microbes indicates that the proteins most similar to AMUC_1438 appear to be found mainly in environmental bacteria. In contrast, the bacterial M60-like *O*-glycopeptidases, encompassing MEROPS families M60, M88, and M98, are found largely in host-adapted bacteria (11). Thus, AMUC_1438 is a member of a large protein family whose biological roles may be diverse.

The CAT region of AMUC_1438 displayed remarkable structural similarity with OgpA (9), including conservation of the metzincin catalytic center and some features of the glycan-binding site. This only translates, however, to ∼19% amino acid sequence identity, which appears to preclude overlapping classification in any existing domain families, other than limited similarity around the metallopeptidase motif. The low sequence identity underpins the failure to retrieve OgpA with BLAST searches using the CAT sequence. Nevertheless, some sequences that displayed ∼30 to 40% sequence identity to CAT were found to also have ∼30 to 40% amino acid sequence identity with OgpA. This points to the evolutionary relationship of the CAT region of AMUC_1438 and OgpA. OgpA itself does not classify into any known MEROPS family and has been suggested to comprise the founding member of a new family. Our analysis suggests that these two proteins form the first characterized members of a very large and amino acid sequence diverse family of peptidases. OgpA and the CAT region of AMUC_1438 would appear to be founding members of potential subfamilies within this novel peptidase family.

The peptidase activity of AMUC_1438 was absolutely dependent on the presence of an α-linked *O*-GalNAc modification, and it would not accommodate a longer glycan. Similar to the M60-like *O*-glycopeptidases and OgpA, the enzyme cleaved immediately N-terminal to the glycosylated residues. We quantified this activity using a custom FRET *O*-glycopeptide. The *k*_cat_ values obtained for the CAT and FL constructs were similar and would be considered quite poor by orders of magnitude compared with the “average” enzyme (24). Similarly, poor turnover on a different *O*-glycosylated FRET substrate was observed for the M60 *O*-glycopeptidase ZmpB from *C. perfringens* (21). At present, it is unclear if this is an inherent property of this class of enzyme, a result of the presence of the FRET pair on the peptide, or an influence of the peptide sequence of the substrate. Indeed, the influence of peptide sequence on the activity of *O*-glycopeptidases in general remains an open question. At present, this has only been systematically investigated for one enzyme and for one position of the peptide substrate. This revealed that the M88 *O*-glycopeptidase IMPa showed a dependence on the nature of the residue in the P1 position (22). Nevertheless, this supports the concept that the amino acid sequence of the substrate is likely important to catalysis by *O*-glycopeptidases. In the case of CAT, it appeared to be completely inactive on a fetuin-based peptide, indicating some selectivity for amino acid sequence and perhaps supporting the concept that the IgA1 hinge sequence used in the FRET substrate may have been a nonoptimal substrate.

The *K*_*M*_ values for the CAT and FL constructs were approximately twofold different. Given the very slow turnover of substrate, the *K*_*M*_ values are good approximations of the substrate-binding affinities (*i.e.*, an approximation of the dissociation constant, *K*_*d*_). The difference between the CAT and FL constructs is the presence of the α-helical linker and putative CBM in FL, which presumably contributed to the increased affinity of the substrate for the enzyme. Consistent with this hypothesis, when the OgpA structure in complex with an *O*-glycopeptide (glycodrosocin) is overlaid with the FL model to approximate the position of an *O*-glycopeptide in the AMUC_1438 active site, the extrapolated path of the peptide from its N terminus extends toward the putative CBM. In the case of the IgA1FRET substrate, this may promote additional nonspecific interactions between the HiLyteFluor 488 group on the N terminus of this substrate. However, for larger natural *O*-glycoprotein/glycopeptide substrates, the trajectory of the substrate may bring the putative CBM-binding site in proximity to additional glycosylation sites, thus promoting multipoint attachment to substrates. The proposed opening and closing domain motions of the enzyme might better accommodate recognition of larger more heterogeneous glycoprotein substrates. This is consistent with the typical functional role of CBMs present in carbohydrate-active enzymes and the proposed role of CBMs in the large multimodular ZmpB *O*-glycopeptidase (21, 25). It is also analogous to the suggested role of a unique praline-binding domain in the IMPa *O*-glycopeptidase (26).

The biochemical activity of AMUC_1438 is consistent with its assignment as an *O*-glycopeptidase that requires the Tn-antigen for substrate recognition and peptide bond hydrolysis. Through this action, it likely assists *A. muciniphila* in this bacterium’s ability to degrade mucin. AMUC_1438, however, is presently unique amongst known *O*-glycopeptidases for its strict specificity for the minimal Tn-antigen and inability to accept larger glycans. All other known *O*-glycopeptidases accept a core 1 *O*-glycan (Galβ-1,3-GalNac, T-antigen) or larger glycan, though they may also be active when only the Tn-antigen is present (12). The biological significance of the strict AMUC_1438 activity is presently unclear, particularly in light of the observation that the other known *A. muciniphila O*-glycopeptidases cleave at sites bearing the Tn-antigen as well as larger glycans (12). The activity of these other *O*-glycopeptidases, however, is only qualitatively known, and it is possible that they are inefficient when only the Tn-antigen is present at a cleavage site. Under these circumstances, the deployment of a specialist such as AMUC_1438 may be beneficial to optimize mucin depolymerization by targeting sites that are poor substrates for the other enzymes. However, in general, the Tn-antigen is relatively rare in healthy tissue, including in MUC2 of the colonic mucin layer, which is a likely substrate for the *A. muciniphila O*-glycopeptidases (27, 28). Therefore, it is likely that the myriad glycoside hydrolases produced by *A. muciniphila* work together as a consortium to trim *O*-glycans on mucins to reveal additional AMUC_1438 cleavage sites. Overall, the function revealed for AMUC_1438 continues to highlight the sophisticated molecular mechanisms underpinning the interaction of *A. muciniphila* with mucin as well as the diversity in *O*-glycopeptidases that is being uncovered.

## Experimental procedures

### Materials

All reagents, chemicals, and other carbohydrates were purchased from Sigma unless otherwise specified.

### Cloning and mutagenesis

Relevant gene fragments encoding the targeted AMUC_1438 protein truncations were amplified by PCR from *A. muciniphila* (American Type Culture Collection; BAA-835) genomic DNA. Specific primer combinations were used to amplify specific gene fragments, as outlined in Table S2. The amplified products were cloned into pET28a using the Takara-Bio In-Fusion cloning kit. The recombinant plasmids encoded the desired polypeptide fused to an N-terminal six-histidine tag by a thrombin protease cleavage site. Mutagenesis of the CAT-encoding gene fragment in pET28a to introduce point mutations was performed using the QuikChange approach (Agilent Technologies). All mutagenic primers are listed in Table S2. The fidelity of all constructs was confirmed by bidirectional sequencing.

### Protein production and purification

Plasmids encoding the desired proteins were transformed into *E. coli* strain BL21 DE3∗. The cells were used directly to inoculate 6 l of 2xYT media supplemented with kanamycin antibiotic (50 μg/ml) and grown at 37 °C while shaking for approximately 5 to 7 h to reach an absorbance of around 0.9 at 600 nm. Protein expression was then induced by the addition of isopropyl-β-d-1-thiogalactopyranoside to a final concentration of 0.5 mM. Cultures were incubated with shaking at 16 °C overnight. Cell cultures were then pelleted by centrifugation at 4 °C.

The cell pellet was resuspended with 15 ml of sucrose solution (25% sucrose, 20 mM Tris–HCl, pH 8.0), prior to adding 10 mg of lysozyme to stir for 20 min. A 30 ml volume of deoxycholate solution (1% deoxycholate, 1% Triton X-100, 50 mM Tris–HCl, pH 8, 100 mM NaCl) was then added. MgCl_2_ was added to a final concentration of 0.5 μM, and 90 μl of DNase I (2 mg/ml) was finally added. The lysed cells were centrifuged (in a Beckman Coulter Avanti J-E) at 16,500*g* for 30 min. The protein of interest was purified from the clarified lysate by loading the supernatant onto Ni^2+^ immobilized metal affinity chromatography resin (GE Healthcare Streamline Chelating beads). The pure fractions were concentrated using a stirred ultrafiltration unit (Amicon) using a 10-kDa membrane (EMD Millipore). The proteins were further purified by size-exclusion chromatography using a Sephacryl S-200 HR column (GE Healthcare) in 20 mM Tris–HCl, pH 8.0, and 500 mM NaCl with 10% glycerol. Selected fractions were again concentrated in a stirred ultrafiltration cell. Concentrations of the proteins were determined by measuring the absorbance at 280 nm and using the specific extinction coefficients for each protein construct (Fig. 1*A*).

### Synthesis of *O*-glycopeptides

A peptide derived from human MUC1 with the sequence GPAPGSTAPPAE was obtained commercially (Bio Basic, Inc) and labeled at its N terminus with BODIPY-FL NHS ester (Lumiprobe Corporation) as described by the manufacturer. All glycosyl transferases were expressed and purified from *E. coli* using a maltose-binding protein fusion expression plasmid described previously (29). The Tn-antigen (GalNAcα1-Thr) and core 1 (Galβ1–3GalNAcα1-Thr) glycan intermediates were synthesized *via* sequential ppGalNAcT2 and core 1 GalT reactions as described previously (21). The Sia2,3core 1 glycan (Neu5Acα2–3Galβ1–3GalNAcα1-Thr) was synthesized in a reaction mixture of 50 mM Hepes (pH 7.0), 1 mM core 1 peptide, 0.1 mg/ml porcine ST3Gal1 (30), and 2 mM CMP-Neu5Ac. Core 2 glycan (Galβ1–3[GlcNAcβ1–6]GalNAcα1-Thr) was synthesized in a reaction mixture of 50 mM Hepes (pH 7.4), 1 mM core 1 peptide, 0.1 mg/ml viral β1,6GlcNAcT, and 2 mM UDP-GlcNAc. The viral β1,6GlcNAcT was expressed as a Δ41 amino acid N-terminal truncation of Bo17 from bovine herpesvirus 4V test strain (31). All reactions were incubated at 30 °C and monitored by HPLC using an Accucore C18 column (3.0 × 100 mm, 2.6 μm; Thermo Fisher Scientific). A Shimadzu Prominence Series HPLC was used with fluorometric detection (Shimadzu RF-20A; excitation 503/emission 514) and a 5 min elution gradient from 20 to 40% acetonitrile (ACN) in 10 mM ammonium acetate (pH 4.5), at a flow rate of 0.6 ml/min and a temperature of 40 °C. The product of each reaction was purified on C18-derivatized silica (Supelco) between synthesis steps with elution in 100% MeOH and then drying before the next step was performed.

### Activity assays

BSM type I-S, bovine fetuin, and bovine asialofetuin were used as general glycoprotease substrates as described (10). Purified enzymes were incubated with substrate in ∼1:200 (w/w) ratio (0.2 μg/ml enzyme and 40 μg/ml substrate) for ∼20 h in 20 mM Tris–HCl, pH 7.5, 0.5 mM ZnCl_2_, at 37 °C. Reactions were then separated on 10% SDS-PAGE gels and stained for specific glycoprotein detection with the periodic acid-Schiff stain (32). The plate-based mucinase assay using biotinylated BSM was performed as described previously (10). Reactions contained 5 μM enzyme in phosphate-buffered saline containing 0.5 mM ZnCl_2_, 1% (w/v) bovine serum albumin, with and without 50 mM EDTA. Reactions were incubated for 18 h at 37 °C.

Detection of peptidase activity on defined *O*-glycopeptides was analyzed by TLC. All samples were separated in a solvent comprising butanol:acetic acid:H_2_O (45:35:30, v:v:v). Unlabeled *O*-glycopeptides were incubated with CAT (10 μM) in 20 mM Tris–HCl (pH 7.5) for 3 h at 37 °C with peptide at 5 μg/μl. Reactions (3 μl each) were spotted onto a silica gel TLC plate. These reactions were developed using ninhydrin solution (1 g in 95 ml pyridine and 5 ml acetic acid) used to develop the TLC plate at 110 °C for 15 min. The BODIPY-labeled peptides at 1 μg/μl were incubated with CAT (1 μM) in 20 mM Tris–HCl (pH 7.5) for 3 h at 37 °C. Reactions (3 μl each) were spotted onto a silica gel TLC plate, and plates were imaged under UV at a wavelength of 365 nm.

A custom FRET-based substrate, referred to as IgA1FRET, was ordered from AnaSpec. The sequence used -TPSP**S**TPPTK- was based on the IgA1 hinge region, where the bold and underlined serine residue bears an α-linked *O*-GalNAc. The N-terminal fluorophore was HiLyteFluor 488, and the C-terminal dark quencher was QXL 520. All steady-state kinetics were performed at room temperature on a SpectraMax M5 plate reader in 384-well microtiter plates using SoftMax Pro 6.2.1 software (Molecular Devices). Standard reaction mixtures were done in 20 mM Tris–HCl (pH 7.0) and 100 μM zinc chloride containing 1 μM of enzyme and 0 to 1000 μM of IgA1FRET. Fluorescence resulting from enzyme activity was measured at 25 ºC using the wavelength of 492 and 530 nm for excitation and emission, respectively, with the addition of a cutoff filter at 515 nm. The HiLyteFluor 488-labeled peptide with the sequence TPSP, the product of hydrolysis, was used to generate a standard curve for product concentration. The measured fluorescence for the activity assays was corrected for inner filter effects for each substrate concentration as previously described (21, 33). Kinetic values for CAT and FL were determined by fitting the Michaelis–Menten equation to the rate data.

### MS

Recombinantly expressed podocalyxin, CD43, and PSGL-1 were purchased from R&D Systems (1658-PD, 9680-CD, and 3345-PS, respectively). C1 esterase inhibitor from human plasma (catalog no.: E0518) and sialidase (catalog no.: 11080725001) were purchased from Sigma. Each protein was reconstituted in 100 ng/μl of 50 mM ammonium bicarbonate. For each protein, four 1 μg samples were prepared. CAT was added to two of the samples at a 1:10 enzyme:protein ratio. Sialidase (100 μU) was added to two samples: one without CAT and one including CAT. The digestion was incubated at 37 °C overnight. Samples were then reduced in 2 mM DTT at 65 °C for 30 min. After cooling, iodoacetamide was added to a concentration of 3 mM and allowed to react for 15 min in the dark at room temperature. Samples were then diluted using 50 μl of 50 mM ammonium bicarbonate. GluC (Promega) was then added to each sample at a 1:20 enzyme:protein ratio and incubated at 37 °C for 6 h. The reaction was quenched using 100 μl of 0.5% formic acid (Sigma) in ultrapure water (Pierce). C18 cleanup was performed using 1 ml strataX columns (Phenomenex). Each column was hydrated with 1 ml of ACN, followed by one time of 1 ml rinse of 0.1% formic acid in water (“buffer A”). The samples were then added to the column and rinsed with 150 μl of 0.1% formic acid. Finally, the samples were eluted twice with 150 μl of 0.1% formic acid in 30% ACN and dried by vacuum centrifugation. The samples were reconstituted in 10 μl of buffer A for MS analysis.

Samples were analyzed by online nanoflow liquid chromatography–tandem MS using an Orbitrap Eclipse Tribrid mass spectrometer (Thermo Fisher Scientific) coupled to a Dionex Ultimate 3000 HPLC (Thermo Fisher Scientific). A portion of the sample (400 ng) was loaded *via* autosampler isocratically onto a C18 nano precolumn using buffer A. For preconcentration and desalting, the column was washed with 2% ACN and 0.1% formic acid in water (“loading pump solvent”). Subsequently, the C18 nano precolumn was switched in line with the C18 nano separation column (75 μm × 250 mm EASYSpray containing 2 μm C18 beads) for gradient elution. The column was held at 35 °C using a column heater in the EASY-Spray ionization source (Thermo Fisher Scientific). The samples were eluted at a constant flow rate of 0.3 μl/min using a 60 min gradient. The gradient profile was as follows: 0-0-35-95-95-2%B in 0-5-65-70-75 to 77 min, respectively.

The instrument method used an MS1 resolution of 60,000 full width at half maximum at 400 *m/z*, an automatic gain control (AGC) target of 3e5, and a mass range from 300 to 1500 *m/z*. Dynamic exclusion was enabled with a repeat count of 3, repeat duration of 10 s, and exclusion duration of 10 s. Only charge states 2 to 6 were selected for fragmentation. MS2s were generated at top speed for 3 s. Higher energy collisional dissociation (HCD) was performed on all selected precursor masses with the following parameters: isolation window of 2 *m/z*, 28% collision energy, orbitrap detection (resolution of 7500), maximum injection time of 75 ms, and an AGC target of 1e4 ions. Electron-transfer/higher energy collision dissociation with supplemental activation was performed if (1) the precursor mass was between 300 and 1500 *m/z* and (2) three of nine HexNAc or NeuAc fingerprint ions (126.055, 138.055, 144.07, 168.065, 186.076, 204.086, 274.092, and 292.103) were present at ±0.1 *m/z* and greater than 5% relative intensity. Electron-transfer/higher energy collision dissociation parameters were as follows: Orbitrap detection (resolution of 7500) calibrated charge-dependent electron transfer dissociation times, 15% normalized collision energy for HCD, maximum injection time of 250 ms, reagent AGC target of 5e5, and precursor AGC target of 1e4.

Raw files were searched using *O*-Pair search with MetaMorpheus against directed databases containing the recombinant protein of interest. Files were searched using nonspecific cleavage specificity. Mass tolerance was set at 10 ppm for MS1s and 20 ppm for MS2s. Cysteine carbamidomethylation was set as a fixed modification, and methionine oxidation was allowed as a variable modification. The default *O*-glycan database was included, and a maximum number of glycosites per peptide was set to 4. Peptide hits were filtered using a 1% false discovery rate. All peptides were manually validated and/or sequenced using Xcalibur software (Thermo Fisher Scientific). After all peptides unique to the mucinase-digested samples were sequenced, peptides ±5 amino acids from the cleavage site were input into weblogo.berkeley.edu to generate the consensus motif.

### Crystallization, diffraction data collection, and processing

All crystals were grown at 18 °C by hanging drop or sitting drop vapor diffusion with 1:1 ratios of crystallization solution and protein. ALT crystals were grown in 0.2 M (NH_4_)_2_SO_4_, 20% (w/v) PEG3350, and 0.1 M Hepes, pH 7.5 with the protein at 63 mg/ml. ALTL crystals were grown in 0.2 M (NH4)_2_PO_4_, 20% (w/v) PEG3350, and 0.1 M bicine, pH 9 with the protein at 20 mg/ml.

Diffraction data were collected on an instrument comprising a Pilatus 200K 2D detector coupled to a MicroMax-007HF X-ray generator with a VariMaxTM-HF ArcSec Confocal Optical System and an Oxford Cryostream 800. Data were integrated, scaled, and merged using HKL2000. Data processing statistics are shown in Table 1.

### Structure solution and refinement

The structure of ALT was determined by the single isomorphous replacement with anomalous scattering method using a native dataset and an iodide derivative. Initial phases were determined using the SHARP/autoSHARP pipeline (34). Phases were improved using PARROT to perform density modification and noncrystallographic averaging (35). An initial model was constructed by autobuilding using BUCANNEER (36). The structure of ALTL was determined by molecular replacement using the ALT model and PHASER (37). The ALTL model was completed by autobuilding using BUCANNEER. Both ALT and ALTL models were finalized by successive rounds of model building with Coot and refinement with REFMAC (38, 39).

For all structures, the addition of water molecules was performed in Coot with FINDWATERS and manually checked after refinement. In all datasets, refinement procedures were monitored by flagging 5% of all observations as “free” (40). Model validation was performed with MolProbity (41). Model refinement statistics are shown in Table 1.

### HDX-MS sample preparation

HDX reactions comparing ALTL with FL proteins were carried out in 20 μl reactions. Reactions contained either 5 μM ALTL (20 pmol, 4 μl) or 5 μM FL (20 pmol, 4 μl). Exchange reactions were initiated by the addition of 16 μl of D_2_O buffer (20 mM Hepes, pH 7.5, 100 mM NaCl, 94.34% D_2_O [V/V]) to 4 μl of protein mixture (final D_2_O concentration of 75.47%). The reactions proceeded for 3, 30, 300, or 3000 s at room temperature, before being quenched with ice-cold acidic quench buffer resulting in a final concentration of 0.6 M guanidine–HCl and 0.9% formic acid post quench. All conditions and time points were created and run in independent triplicate. Samples were flash frozen immediately after quenching and stored at −80 °C until injected onto the ultraperformance liquid chromatography (UPLC) system for proteolytic cleavage, peptide separation, and injection onto a QTOF for mass analysis, described later.

### Protein digestion and MS/MS data collection

Protein samples were rapidly thawed and injected onto an integrated fluidics system containing a HDx-3 PAL liquid handling robot and climate-controlled (2 °C) chromatography system (LEAP Technologies), a Dionex Ultimate 3000 UHPLC system, as well as an Impact HD QTOF Mass spectrometer (Bruker). The full details of the automated LC system are as previously described (42). The protein was run over one immobilized pepsin column (Trajan; ProDx protease column, 2.1 mm × 30 mm PDX.PP01-F32) at 200 μl/min for 3 min at 8 °C. The resulting peptides were collected and desalted on a C18 trap column (Acquity UPLC BEH C18 1.7 mm column (2.1 × 5 mm); Waters; catalog no.: 186003975). The trap was subsequently eluted in line with an Acquity 1.7 μm particle, 100 × 1 mm^2^ C18 UPLC column (Waters), using a gradient of 3 to 35% B (buffer A 0.1% formic acid; buffer B 100% ACN) over 11 min immediately followed by a gradient of 35 to 80% over 5 min. MS experiments acquired over a mass range from 150 to 2200 *m/z* using an electrospray ionization source operated at a temperature of 200 °C and a spray voltage of 4.5 kV.

### Peptide identification

Peptides were identified from the nondeuterated samples of ALTL and FL using data-dependent acquisition following tandem MS/MS experiments (0.5 s precursor scan from 150 to 2000 *m/z*; 12 0.25 s fragment scans from 150 to 2000 *m/z*). MS/MS datasets were analyzed using PEAKS7 (PEAKS), and peptide identification was carried out by using a false discovery–based approach, with a threshold set to 0.1% using a database of purified proteins and known contaminants. The search parameters were set with a precursor tolerance of 20 ppm, fragment mass error of 0.02 Da, charge states from 1 to 8, leading to a selection criterion of peptides that had a −10logP score of 34.8 and 32.7 for ALTL and FL, respectively.

### Mass analysis of peptide centroids and measurement of deuterium incorporation

HD-Examiner Software (Sierra Analytics) was used to automatically calculate the level of deuterium incorporation into each peptide. All peptides were manually inspected for correct charge state, correct retention time, appropriate selection of isotopic distribution, and so on. Deuteration levels were calculated using the centroid of the experimental isotope clusters. Results are presented as relative levels of deuterium incorporation, and the only control for back exchange was the level of deuterium present in the buffer (75.47%). Differences in exchange in a peptide were considered significant if they met all three of the following criteria: ≥4.5% change in exchange, ≥0.45 Da difference in exchange, and a *p* value <0.01 using a two-tailed Student's *t* test. The raw HDX data are shown in two different formats. The raw peptide deuterium incorporation graphs for a selection of peptides with significant differences are shown in Fig. S5, with the raw data for all analyzed peptides in the source data. To allow for visualization of differences across all peptides, we utilized number of deuteron difference (#D) plots (Fig. 7*C*). These plots show the total difference in deuterium incorporation over the entire H/D exchange time course, with each point indicating a single peptide. Samples were only compared within a single experiment and never compared with experiments completed at a different time with a different final D_2_O level. The data analysis statistics for all HDX-MS experiments are in Table S3 according to guidelines (43).

## Data availability

The atomic coordinates for the two crystal structures reported here have been deposited in the Research Collaboratory for Structural Bioinformatics Protein Databank (www.rcsb.org) under the accession codes 8DF2 and 8DEK. The MS proteomics data have been deposited to the ProteomeXchange Consortium *via* the PRIDE partner repository (44) with the dataset identifier PXD034904.

## Supporting information

This article contains supporting information.

## Conflict of interest

The authors declare that they have no conflicts of interest with the contents of this article.
