# Supplementary material for: A previously uncharacterized O-glycopeptidase from Akkermansia muciniphila requires the Tn-antigen for cleavage of the peptide bond
Source: J Biol Chem. 2022 Aug 30;298(10):102439. doi: 10.1016/j.jbc.2022.102439 (PMC9513282; doi:10.1016/j.jbc.2022.102439)
Supplement: Supporting Information [file mmc1.pdf]

Supporting Information

A previously uncharacterized *O*-glycopeptidase from *Akkermansia muciniphila* requires the Tn antigen for cleavage of the peptide bond.

Brendon J. Medley[1], Leif Leclaire[2], Nicole Thompson[2], Keira E. Mahoney[3], Benjamin Pluvinage[1], Matthew A.H. Parson[1], John E. Burke[1,4], Stacy Malaker[3], Warren Wakarchuk[2], and Alisdair B. Boraston[1]*.

[1]Department of Biochemistry and Microbiology, University of Victoria, PO Box 1700 STN CSC, Victoria, British Columbia, V8W 2Y2, Canada. [2]Department of Biological Sciences, University of Alberta, Edmonton, AB T6G 2E9, Canada. [3]Department of Chemistry, Yale University, 350 Edward St., New Haven CT, 06511. [4]Department of Biochemistry and Molecular Biology, University of British Columbia, Vancouver, British Columbia, V6T 1Z3, Canada.

Running title: *A metzincin-like O-glycopeptidase*

*To whom correspondence should be addressed: Alisdair B. Boraston, Department of Biochemistry and Microbiology, University of Victoria, Victoria, British Columbia, Canada, V8P 5C2; boraston@uvic.ca; Tel. +1 (250) 472-4168; Fax. +1 (250) 721-8855

Table S2: Oligonucleotide primers and the constructs they were used for.

Primer Name	Oligonucleotide sequence
ALT_Fwd	5’-CAGCCATATGGCTAGCATGGATGAGGAGTCAGCTTCCAGGGCATCTGTTCCTGCC-3’
ALT_Rev	5’-GGTGGTGGTGCTCGAGTTACTGGCGCCCCAGAGCGCGCTTCCAG-3’
ALTL_Rev	5’-GGTGGTGGTGCTCGAGTTAGGTTGCCGCAGGAATCTGCGCCGG-3’
PEP_Fwd	5’-CAGCCATATGGCTAGCGGCAAACCGAAGTCTTCCAGAAAATTGCATGTGGTTTACGTAAC-3’
CAT_Fwd	5’-CAGCCATATGGCTAGCGACAGGGAGGGAGCGG-3’
CAT_Rev	5’-GGTGGTGGTGCTCGAGTTATCCCCGGGCATCCATCC-3’
CBM_Fwd	5’-CAGCCATATGGCTAGCGCCAGTATCAGCCTGAATGACTGCAAGCCTTCCG-3'
CBM_Rev	5’-GGTGGTGGTGCTCGAGTTACCGGGTCAGCATGCCGTTGGCTATAATGCCCC-3’
E209Q_Fwd	5’-GCCCATCAATTGGGGCATTCCTTCG-3'
E209Q_Rev	5’-CCCCAATTGATGGGCCGTACCG -3'
Y202A_ Fwd	5'-GAAAGAACGCCACCATTGCCATAGGCGGTACGGCCC-3'
Y202A_ Rev	5'-GGGCCGTACCGCCTATGGCAATGGTGGCGTTCTTTC-3'
E208A_ Fwd	5'-CGGTACGGCCCATGCATTGGGGCATTCCT-3
E208A_ Rev	5'-AGGAATGCCCCAATGCATGGGCCGTACCG-3'
D151A_ Fwd	5'-CCAGCTTCCGGCCGGCGTGGGTC-3’
D151A_ Rev	5'-GACCCACGCCGGCCGGAAGCTGG-3'
Y156A_ Fwd	5'-CGGCGTGGGTCCTGCTTACGGCGGCGGC-3'
Y156A_ Rev	5'-GCCGCCGCCGTAAGCAGGACCCACGCCG-3’

Construct	Forward primer	Reverse primer
ALT	ALT_Fwd	ALT_Rev
ALTL	ALT_Fwd	ALTL_Rev
PEP	PEP_Fwd	ALT_Rev
PEPL	PEP_Fwd	ALTL_Rev
CAT	CAT_Fwd	CAT_Rev
CBM	CBM_Fwd	CBM_Rev
FL	ALT_Fwd	CBM_Rev

Table S3. HDX Data Summary

Protein Data Set	ALTL	FL
HDX reaction details	%D2O=75.47%<br>pH(read)= 7.5<br>Temp= 18°C	%D2O=75.47%<br>pH(read)= 7.5<br>Temp= 18°C
HDX time course	3s, 30s, 300s, 3000s	3s, 30s, 300s, 3000s
HDX controls	N/A	N/A
Back-exchange	Corrected based on %D2O	Corrected based on %D2O
Number of peptides	124	124
Sequence coverage	94.2%	94.2%
Average peptide length / Redundancy	Length = 17.8<br>Redundancy = 4.6	Length = 17.8<br>Redundancy = 4.6
Replicates	3	3
Repeatability	Average StDev = 0.5%	Average StDev = 0.5%
Significant differences in HDX	>5% and >0.5 Da and unpaired t-test <0.01	>5% and >0.5 Da and unpaired t-test <0.01

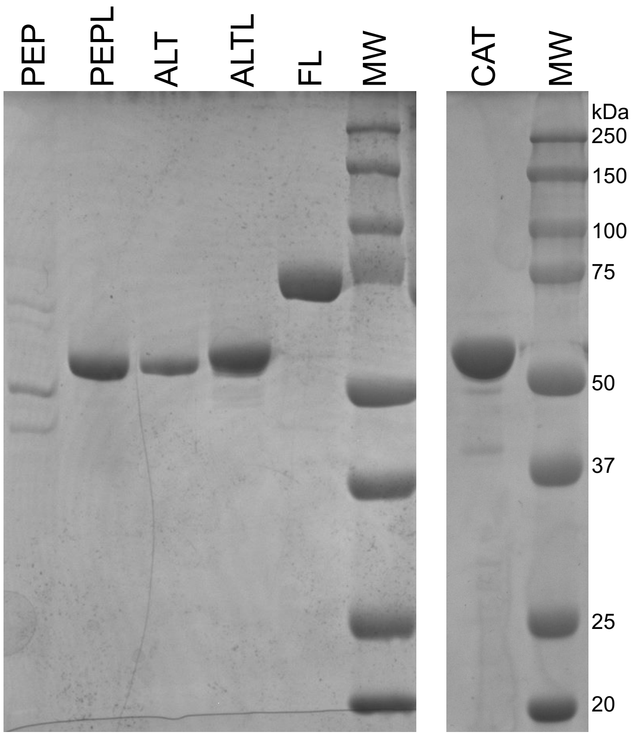

Figure S1. SDS-PAGE analysis of proteins used in this study.

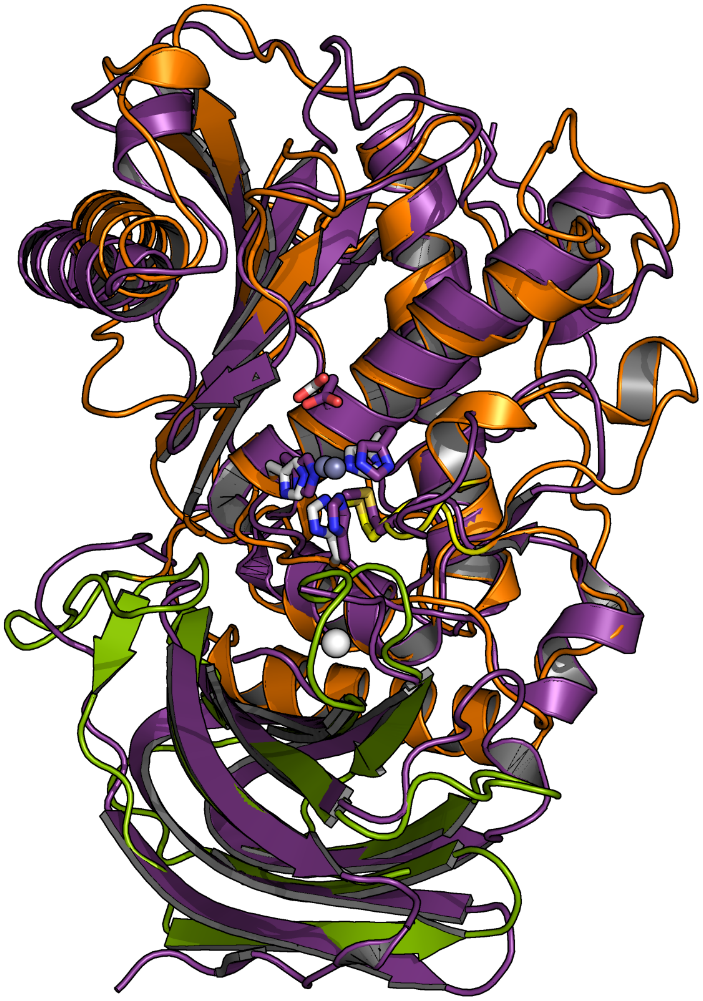

Figure S2. Overlap of the ALT structure (orange, green, yellow, and grey) and OgpA (purple, 6Z2O).

A

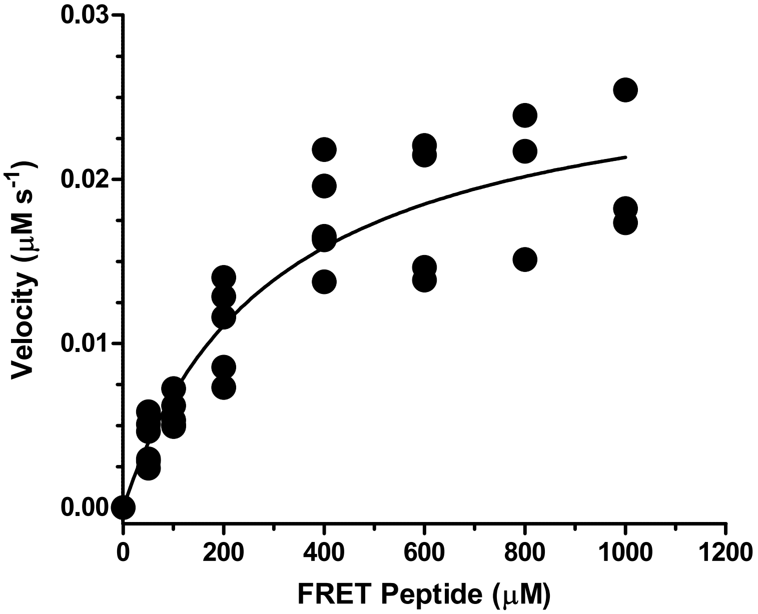

B

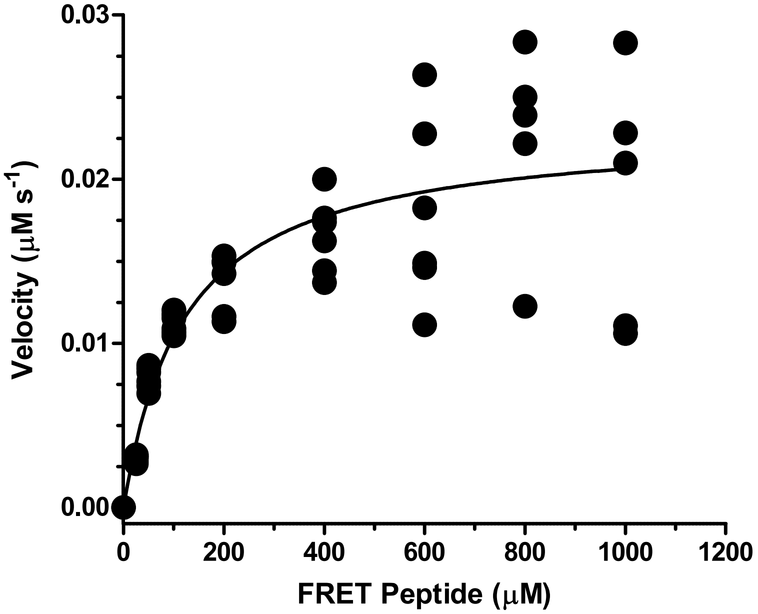

Figure S3. Individual replicates of kinetic data on the IgA1FRET substrate for A) CAT and B) FL.

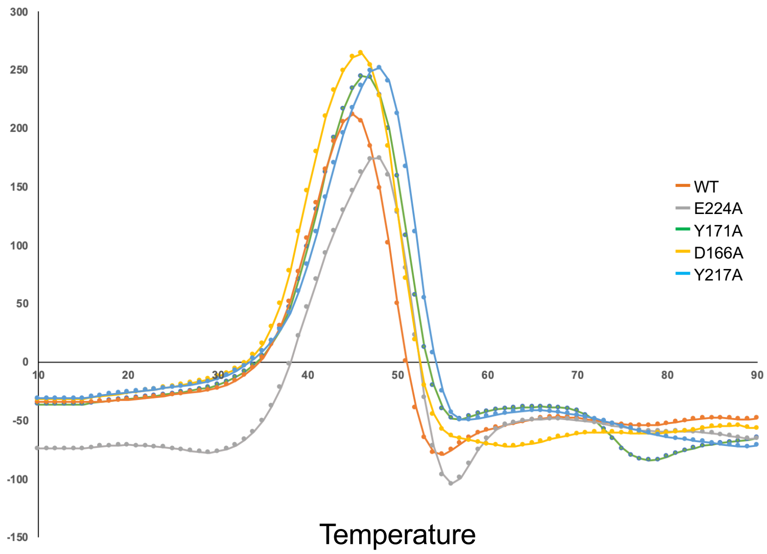

Figure S4. First derivative of thermal melting curves performed by differential scanning fluorimetry. The melting temperatures, Tm, are approximated by the peaks, which are within approximately ±2 ºC, indicating all of the proteins are folded.

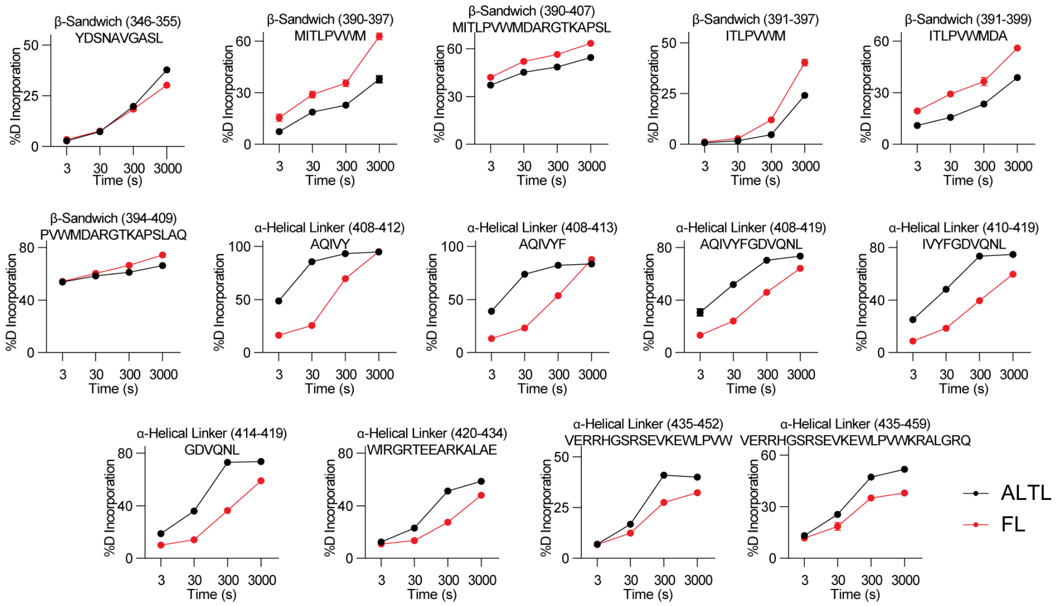

Figure S5. Deuterium incorporation difference for all peptides that showed a significant increase or decrease in exchange (>5%, 0.4 Da, and an unpaired t-test P<0.01). For all panels, error bars show SD (n = 3).

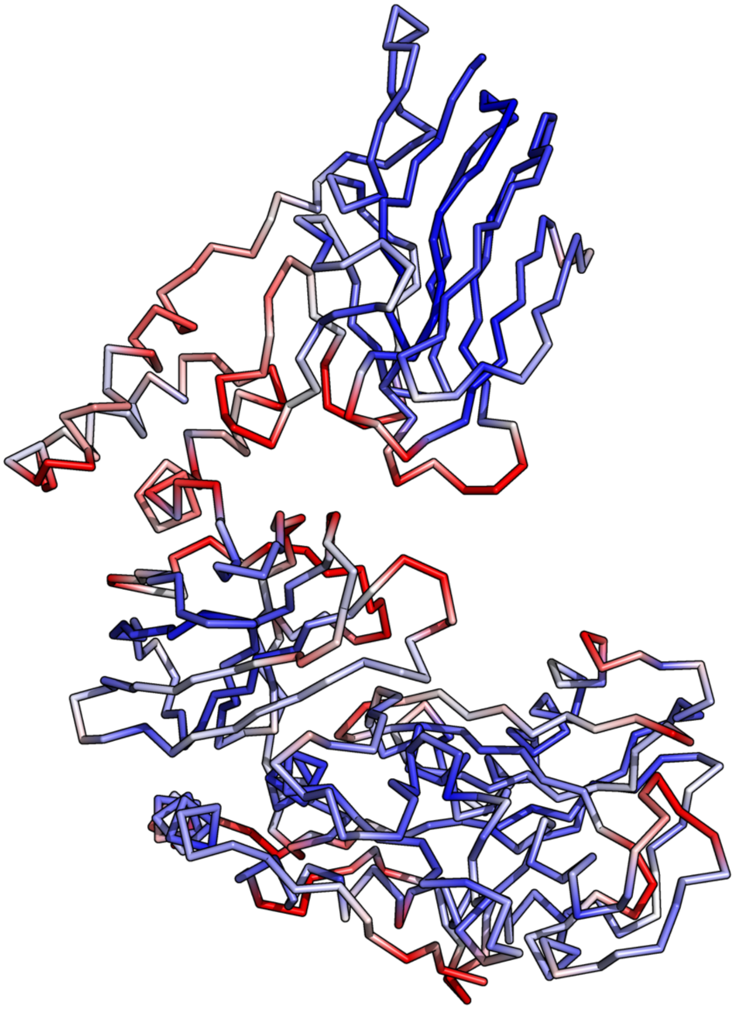

Figure S6. Deformability of the FL model as determined by normal mode analysis using iMODS. Deformability is color-ramped from low deformability to high deformability as blue-white-red.
